# Understanding the Antecedents of Organic Food Consumption in Pakistan: Moderating Role of Food Neophobia

**DOI:** 10.3390/ijerph16204043

**Published:** 2019-10-22

**Authors:** Ahsan Akbar, Saqib Ali, Muhammad Azeem Ahmad, Minhas Akbar, Muhammad Danish

**Affiliations:** 1 International Business School, Guangzhou College of South China University of Technology, Guangzhou 510080, China; akbar@gcu.edu.cn; 2 Department of Management Sciences, COMSATS University Islamabad (Sahiwal Campus), Sahiwal 57000, Pakistan; minhasakbar@cuisahiwal.edu.pk (M.A.); m.danish33@outlook.com (M.D.); 3 Department of Management Sciences, Barani Institute of Sciences Sahiwal, Sahiwal 57000, Pakistan; azeem@baraniinstitute.edu.pk

**Keywords:** organic food, food neophobia, Green Perceived Value model

## Abstract

Environmental and health problems have increased the interest of researchers and practitioners in investigating the factors that affect organic food consumption. However, little attention has been paid to the actual organic food buying behavior, particularly in developing countries like Pakistan. Therefore, the aim of the present study is to determine the actual buying patterns of consumers. For this purpose, a conceptual model based on green perceived value framework which predicts consumer’s purchase intention and purchase behavior has been empirically tested. Likewise, moderating role of food neophobia has also been explored. Data is collected from millennials that are under rated but constitute the most important consumer segment in Pakistan. Structural Equation Modeling (SEM) is employed to analyze the data. Results from 221 university students reveal that functional value, social value, emotional value, and conditional value positively influence the consumer purchase intention. Moreover, purchase intention is positively linked to the consumer purchase behavior of organic food. Furthermore, the study findings also confirm the moderating role of food neophobia between purchase intention and consumption of organic food. This paper depicts some noteworthy insights of consumer behavior for organic food producers, marketers, and researchers. At the end, limitations and recommendations for future research are elaborated.

## 1. Introduction

Environmental deterioration and its negative impacts on human health have become a significant concern for academics and organizations nowadays [1]. Industrialization and economic growth have caused over-consumption, which creates many environmental problems (soil, air, water), deterioration of natural resources, depletion of the ozone layer and health hazards [2,3]. These negative impacts on the environment and ecological imbalances make consumers conscious about the consequences of their actions on the environment and human health [4]. In their search for the solution for these adverse effects, consumers are changing their food consumption patterns [5]. Issues like bird flu, mad cow disease, foot, and mouth epidemics, use of pesticides, toxic chemicals, and other biological ingredients to increase per acre yield have created anxiety among consumers about what they eat and its impact on their health and environment [6]. As a result, the consumption of organic foods has seen phenomenal growth in the recent past.

Organic food is produced through organic agricultural techniques and without the use of conventional methods like ionizing radiation or bioengineering, fertilizers made from sewage sludge or synthetic ingredients [7]. Non-use of chemical pesticides makes it possible for organic products to consume 40% less energy to produce, and support the welfare of animals, soil and the environment [8]. Organic food consumers consider it a healthier choice, to have better taste, and be fresher than conventional food products [9]. Moreover, as per the latest statistics, Austria, Argentina, China, USA, and Spain hold the top five positions in terms of organic land utilization in 2016 [10]. Due to its perceived benefits, there has been exceptional growth in the sale of organic foods worldwide. The worldwide sales grew from 15$ billion in 1999 to 90$ billion in 2016 with a six-fold growth rate. In this sale, US market has the largest share, surging from 3.4$ billion in 1999 to 45$ billion in 2017, while the EU stood second, with a sales volume of 33.5$ billion euros in 2016 [8]. Although most of the organic food consumption occurs in developed countries (90% of overall consumption) most organic food is produced in developing countries, especially in South Asian countries [11].

In the Asian region, organic product producers have increased exponentially from 2015 to 2016 and are expected to grow by 370% in the next 10 years, highest than any region [10]. Pakistan is an economy in South Asia having a 20% contribution of agriculture to the overall GDP. Pakistan is a country which has a 45299-hectare organic area from a total cultivated area of 22.68 million hectares with 111 commodity producers, but has only a 0.1% contribution to the global organic industry [10], [12]. Furthermore, despite the benefits of organic foods and their potential, organic food adoption is quiet low in Pakistan [13,14], while Pakistan’s households spend half of their income on food products, the highest among 84 countries surveyed by the US Department of Agriculture’s Economic Survey. Average Pakistani consumers spent 47.7% of their income on food, as compared to the US where only 6.6% of income was spent on food [15]. Consuming unhealthy foods instead of healthy organic food that can prevent non-communicable diseases (NCDs) is a behavioral issue in Pakistan [16] and they do it for the recreational experience, fun and enjoyment [17]. Reports suggest that the overall risk of NCDs is 56% of the total disease burden in Pakistan. Nevertheless, 60% of deaths in Pakistan are caused by diabetes, cardiovascular disease, hypertension and a variety of cancers [18]. Moreover, the highest proportion of diet related cardiovascular deaths are recorded in Pakistan [19]. The above mentioned stats suggest that there is a need to identify the factors that can predict consumers’ buying behavior of organic food in health-affected countries like Pakistan.

Similarly, identifying factors favoring organic food consumption is also important for marketers to better understand consumers’ motives behind their consumption patterns and to design marketing strategies to increase sales [20]. Prior research has identified several key factors associated with intentions to purchase organic food, e.g., health consciousness, price, quality, notations, taste, food safety, and availability [21,22]. Despite some valuable previous studies, there are still three gaps identified by researchers. Firstly, little attention has been paid to millennials in organic food consumption especially no study has considered millennials in Pakistan which are one of the most important segments in organic food consumption. Second, prior studies have not examined actual organic food buying behavior, especially using the Green Perceived Value (GPV) model [23]. Third, Kushwah et al. [24] conducted a review and concluded that very few moderating variables are used in the context of organic food consumption research. Moreover, no prior study has checked moderating role of food neophobia on intention-behavior relationship while it can be the main cause of low organic food adoption in Pakistan.

Millennials are a very crucial segment for marketers because, with all financial constraints, they are motivated to engage in green consumption [25] in any country, especially Asian countries like China, India, and Pakistan which have relatively more young people than others [26]. Pakistan is declared as one of the youngest countries in the world and the second youngest country in South Asian countries after Afghanistan [27]. This makes Pakistan an important segment for green products e.g., organic food. however, it is also found that teenagers are spending the biggest chunk of their budget on junk food in Pakistan [16]. Keeping this in mind, this study to understand the factors of organic food consumption is focused on millennials.

Consumer purchasing behavior includes decisions regarding products and services which the consumer intends to buy over time. These intentions differ for different product categories, especially for organic (green) products because consumers who buy organic products have different motives than those who buy conventional products [28]. These motives can be well explained by the GPV model which explains why consumers prefer to buy or not to buy green products and why consumers prefer a specific brand over others. In the green marketing literature and environmental research, GPV is considered an influential factor to determine organic food consumption [23]. GPV is defined as “a consumer’s overall appraisal of the net benefits of a product or service between what is received and what is expected based on the consumer’s environmental desires, sustainability expectations, and green needs” [29]. GPV helps to understand consumer needs, expectations, and desires from green products during the decision-making process which cannot be explained by a uni-dimensional concept. Due to complex nature of GPV, Sangroya and Kumar [30] developed a multidimensional GPV model which asserts that consumer intention is predicted by four green perceived values, i.e., functional, social, emotional, and epistemic values. These values predict the consumers’ intentions to consume the specific green product, in this case, organic products. However, to the best of our knowledge, none of the studies have investigated the consumer perspective regarding organic food by employing the GPV model, particularly in a developing country like Pakistan [4,11,14,31,32]. Although the intentions predicted by GPV subsequently lead to the actual buying behavior of a specific product, Woo and Kim [23] contend that it is necessary to check the actual buying behavior of green food by using GPV model.

Furthermore, the previous literature also suggests that intentions are good predictors of behavior but there is often a significant gap between intentions and behavior. One of the underlying reasons for low organic food adoption or this intention-behavior gap could be food neophobia. Food neophobia refers to consumers’ refusal or reluctance to try new, unfamiliar and novel foods [33]. For unfamiliar foods like organic food, consumers’ intentions and behavior are affected by food neophobia. As Schickenberg et al. [34] claim, consumers with food neophobia are unwilling to try healthy foods. Past researchers have checked the relationship of food neophobia in different food categories [35,36], in different cultural contexts [37] and in different age groups, but the moderating role of food neophobia in organic food consumption has not been examined, particularly in Pakistan where organic products consumption is fairly low. Hence, this study aims to fill this gap by finding the antecedents of consumer intentions by using the GPV model and the moderating effect of food neophobia in consumer intention and consumers’ organic food buying behavior in Pakistan.

## 2. Literature Review and Hypothesis Development

### 2.1. Millennials and Green Consumption

The demographic characteristics of consumers are important elements for marketers to design marketing strategies. Age plays an essential role in defining consumer preferences as consumers with similar age brackets usually share common values and consumer behavior. Therefore, taking generation rather than age is a more effective criterion for market segmentation as they undergo the same historical, cultural, political, economic and social events in their life which impact their behavior [38]. Millennials, also referred as generation Y, born between 1982 and 2000, have attracted the attention of marketers, managers, and researchers [39]. Millennials are the most sensitive generation of this era. They care about the environment, health, social values, worry about sustainability and their income [32,40,41]. This young segment is innovative, expresses concern about the future, and they are the future of the world [42]. This generation is characterized by the high usage of technology, internet, and social media, seek higher education, display high efforts towards community and social activities, green products, and particularly food safety and sustainability [40,41,43,44]. In the USA, millennials are progressively involved in organic food consumption. They are more knowledgeable about organic food, willing to pay a premium price and have higher trust in organic labelling [45]. However, south Asian countries have relatively more younger people, and there are only a few studies which consider millennials for organic food consumption [32,46] and specifically none of the studies have considered Pakistan’s millennials’ organic food consumption. Pakistan has now more young people than it ever had and is considered as one of the youngest countries in world. About 64% of the population is younger than 30 years and about 29% of the population lies between the ages of 15 and 29 [27]. Therefore, there is a need to investigate this generational phenomenon to better understand consumers’ consumption behavior of organic food in Pakistan.

### 2.2. Green Perceived Value Model

Perceived value refers to consumers’ overall evaluation of products’ net benefits and utility based on consumers’ appraisal [47]. It is basically a subjective construct which is measured by consumers based on different features of the products. It is considered a key determining factor of consumer behavior [48] and has a positive effect on consumers’ behavior [49]. The perceived value is considered an important factor both from consumer and industrial perspective and several dimensions (utilitarian and hedonic) of perceived values are also suggested by several researchers [50,51,52,53,54,55].

In green marketing and environmental research, Chen and Chang [29] suggested a uni-dimensional Green Perceived Value (GPV) model to understand consumers’ green purchase intentions. GPV refers to the consumers’ overall appraisal of products and services based on green needs, environmental desires, sustainable expectations and the ultimate value received from these products. However, although this uni-dimensional GPV demonstrates well the consumer green intentions but the complexity and multidimensional nature of GPV remains unexplained [23,30,56]. Thereupon, Sangroya and Kumar [30] developed a multidimensional GPV model having four sub constructs (functional value, social value, emotional value, and conditional value). This multidimensional GPV model is based on utilitarian and hedonic benefits and found to be reliable and robust [30]. The four dimensions have a significant effect on consumers intention to buy organic food [23] but the study was limited to purchase intentions. Some researchers have also suggested that there is an intention-behavior gap, so this study also checks the actual buying behavior of organic food as suggested by [23]. Notwithstanding this, due to the low adoption of organic food in Pakistan, the study also examines the moderating role of food neophobia in the intentions-behavior relationship.

### 2.3. Functional Value

Function value refers to the perceived utility obtained from the silent features or utilitarian benefits of a product like functionality, performance, durability, dependability, price, and quality [55]. It is the basic value the consumer desires from any product. In the organic food context, price and quality of the products are very important features. Use of toxic material, animal residues, pesticides, and food additives have increased heath concerns among the consumers [6,57]. Consumers are changing their preferences from conventional foods to organic foods due to health consciousness. Empirically, health concerns are found to be an important factor that influences consumers’ decisions regarding organic food [6,58,59,60]. Testa et al. [61] also confirmed that health consciousness influences consumer attitude to buy organic products.

Price is a very crucial factor because the price of organic foods is higher than conventional foods [59]. However, Padel and Foster [62] argue that consumers are willing to pay a premium whenever they feel the purchase of organic food is justified in terms of its attributes. Still, price is an important feature of organic products because the consumer wants economic value along with quality. Extant research has found a positive influence of functional price value on intentions to buy organic products [2,6,63]. Thus, based on previous studies, the following can be hypothesized:

**Hypothesis 1** **(H1):** Functional value has a significantly positive effect on consumers’ intention to buy organic food.

### 2.4. Social Value

Social value is the perceived utility driven through an individual’s association with one or more distinctive social groups while choosing a product [55]. It is basically related to social identity and self-image of consumers developed in specific groups like friends, family, and peers. Yoo et al. [64] found that consumers like to engage in green consumption behavior due to reasons beyond its functionality which involve their symbolic identification and value by the society. Moreover, consumers who consume green products motivate others to consume such products for environmental protection [65]. Finch [66] suggested that organic product buyer’s behavior differ due to social values. Recent studies found that subjective norms and social values positively influence the consumers’ intention to buy organic products [2,6] but few studies could not found any relationship of social value and green consumption behavior [67,68]. Hence, it can be hypothesized that:

**Hypothesis 2** **(H2):** Social value has a significant and positive effect on consumers’ intention to buy organic food.

### 2.5. Emotional Value

Emotional value is the perceived utility acquired from the association of emotions, feelings and different affective states while choosing between alternative products [55]. These consumer emotions (positive or negative) vary in situations and among individuals which influence consumption behavior. Past experiences regarding product usage predict consumers’ future emotions and ultimately purchase intentions [61]. Consumers experience positive emotions like feeling good, satisfied, well-being and comfort while choosing or consuming green products over conventional ones. Recently it is established that these emotional values lead toward organic food consumption [61]. However, these findings are also verified by previous researches on green consumption behavior [68,69,70] but also have contradictions in green consumption [3,65,71]. So, in the organic food domain, it can be hypothesized that:

**Hypothesis 3** **(H3):** Emotional value has a positive and significant effect on consumers’ intentions to buy organic food.

### 2.6. Conditional Value

Conditional value refers to “the perceived utility acquired by an alternative as the result of the specific situation or set of circumstances facing the choice maker” [55]. It is basically the perceived value attain by some extrinsic situation that can be economic, environmental and physical circumstances that consumers face at the time of purchase [72]. At the time of purchase, the consumer faces different situations, like time, place, discount, and promotions that affect consumer decision makings and choice behavior [73]. Even same situation at different times can result in different value due to past experience of the consumers [74]. Lin and Huang [68] suggested that different incentives, promotional discounts, and subsidies enable the consumers to involve in pro-environmental behavior. Consumer preferences change whenever situations change. Wen and Noor [69] also argue that cash rebate and government subsidy might drive consumer intentions to use green products. Previous researchers also found positive influence of conditional value on green purchase behavior [3,68,75] while some studies could not find any influence on green consumption [71,76,77]. Thus, there is a need for more clarification. Therefore, the following hypothesis is developed:

**Hypothesis 4** **(H4):** Conditional value has a positive and significant effect on consumers’ intentions to buy organic food.

### 2.7. Consumer Purchase Intention and Behavior towards Organic Food

Intentions refer to what extent an individual is willing to perform a certain behavior and tells how many times a person tries to perform a definite behavior [78]. Humans are considered as rational actors, they plan to attain a specific goal and then perform accordingly which means human behavior is shaped by the intentions. Hence, intention to purchase a specific product can result in the adoption of that product or buying behavior of consumers. However, there can be a mismatch between intentions declared by the consumers and what is actual behavior of the consumer at the time of purchase [79,80] which is referred as intention-behavior gap. This intention behavior gap has been identified by different researches on sustainable and green consumption behavior [61,81,82,83].

This discrepancy of intention-behavior gap is also prevalent in organic food consumption. It is also found that consumers tend to overestimate their organic food consumption and a significant proportion of consumers declare that they buy organic food at least once in a month but in actual fact they didn’t buy it at all [61]. Even there is an intention-behavior gap, limited studies exist on actual buying behavior of organic food and prior researches emphasize to ascertain consumers’ actual buying behavior [23,32,84,85]. As without intention, it is not possible to asses consumer buying behavior of a specific product [86,87]. So, based on the above discussion the following hypothesis emerges:

**Hypothesis 5** **(H5):** Purchase intention has a positive and significant effect on consumers’ organic food buying behavior.

### 2.8. Moderating Effect of Food Neophobia

Food neophobia is defined as the reluctance, unwillingness or refusal to consume novel foods, unfamiliar foods and is considered to be the important factor to detect human food consumption behavior [33]. This personality trait has a negative relation with food choices whether it be unfamiliar foods, novel foods, or sometimes familiar foods [88,89] which is evidenced by the study results showing that persons with higher food neophobic personality traits are less likely to consume dietary varieties as compared to their less neophobic counterparts [37]. It is also apparent that consumers fear consuming organic food due to a lack of knowledge about healthier processing or advancements in food technologies [90]. In this regard knowledge plays a very important part in reducing the fear of consumers regarding new food innovations [91].

Likewise, consumers are unwilling to adopt healthy foods; such as functional foods in China due to food neophobia which is totally the opposite in Germany [36], whereas, food neophobic personality was not a significant predictor of organic food consumption in Finland [92]. Some studies suggest direct effect of food neophobia on behavior [36,37,90,93,94] others found an indirect effect of food neophobia on consumers intentions [35,84,89].

It is an established fact that organic food consumption is low in Pakistan [13] and there is a gap between intentions and the actual behavior of consumers regarding organic food consumption [4,14,23]. Studies also observed that intention-behavior relationship can be strengthened by involving moderating variable [95]. Therefore, food neophobia can play a moderating role in purchase intention and actual buying behavior of organic food in Pakistan. Based on the above arguments, it is hypothesized that:

**Hypothesis 6** **(H6):** Food neophobia moderated the relationship between organic food purchase intentions and actual buying behavior.

### 2.9. Theoretical Framework

**Figure 1:**
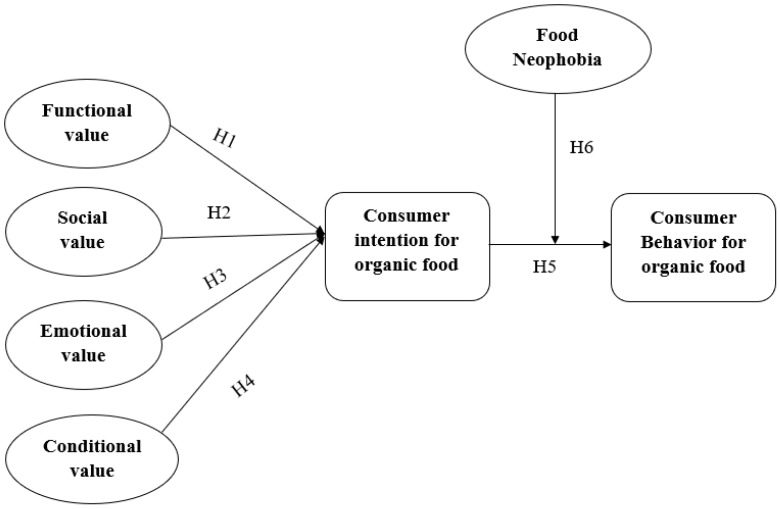
Theoretical Framework.

Based on the above hypothesis a theoretical framework was developed by taking Green Perceived Value as independent variable, its impact on consumer intention which consequently affects their behavior towards organic food. Food neophobia moderates the relationship of purchase intention and behavior for organic food (Figure 1).

## 3. Materials and Methods

The study has been conducted in Pakistan to check the actual buying behavior of millennials regarding organic food consumption. For this purpose, a quantitative approach is adopted, and data was collected from university students of two metropolitan cities (Faisalabad and Sahiwal). Blichfeldt and Malene [96] argue that the university students are at a developing stage and they take steps which are suitable to their own lifestyle. Moreover, these young consumers are considered appropriate for this research due to their distinguishing characteristics like care for environment, food sustainability, and green consumption [32]. The data was collected from University of Sahiwal, COMSATS University Islamabad-Sahiwal campus, University of Agriculture Faisalabad and Government College University Faisalabad. It was difficult to reach all departments, so this study encompasses students from management sciences departments of the universities by using non-probability (purposive sampling) technique. Non-probability sampling is appropriate when it is problematic to asses complete sample frame. In this regard, Calder et al. [97] recommend that non-probability sampling is also appropriate for theoretical generalizability. Roscoe et al. [98] suggested that for behavioral studies, sample size between 30 to 500 would be appropriate. To achieve acceptable sample size, 400 questionnaires were distributed as Nulty [99] observed that normal response rate in consumer studies is around 40% to 60%. A total of 260 questionnaires were returned. After screening, 221 questionnaires were found usable for data analysis, indicating a 55.25% response rate. The response rate is also in line with the finding of Mellahi and Harris [100] who suggested that the average response rate in subcontinent countries like India and Pakistan is 52.68%.

### Measurement Scale

The questionnaire was distributed in two sections. The first section includes demographic information of the respondents (see Table 1). While second section includes the exogenous and endogenous contracts which were adopted from previous researches (see Appendix A). Seven-point Likert scale ranging from strongly disagree = 1 to strongly agree = 7 was used to answer each item of constructs. Four items of functional value were adapted from Biswas and Roy [3]; four items of social value were adapted from Rahnama and Rajabpour [76]; three items of emotional value were adapted from Lin and Huang [68]; four items of conditional value were adapted from Rahnama and Rajabpour [76]; the four items of purchase intention regarding organic food were adapted from Li and Zhong [101]. Only endogenous variable i.e., consumer actual buying behavior was measured by four items that were adapted from Lin and Huang [68]. The five items of moderating variable i.e., food neophobia were adapted from Huang et al. [35].

## 4. Analysis

Structural Equation Modeling (SEM) is used for data analysis. SEM is an influential multivariate second generation data analysis technique having several benefits over conventional multivariate data analysis techniques in terms of efficiency, convenience, and accuracy [102,103]. SEM is of two types i.e., covariance SEM (CB-SEM) and variance SEM (VB-SEM) [104]. Studies suggest the use of PLS-SEM (VB-SEM) to overcome the data normality issues that normally appears in social sciences studies [105,106]. Two stage analysis approach i.e., assessment of measurement and structural model was employed by using Smart PLS 3.0 [104,107]. Validity and reliability is assessed in measurement model and hypotheses are tested in the structural model. Bootstrapping approach (5000 re-sample) is employed to measure the significance of path coefficients [108].

### 4.1. Measurement Model

**Figure 2:**
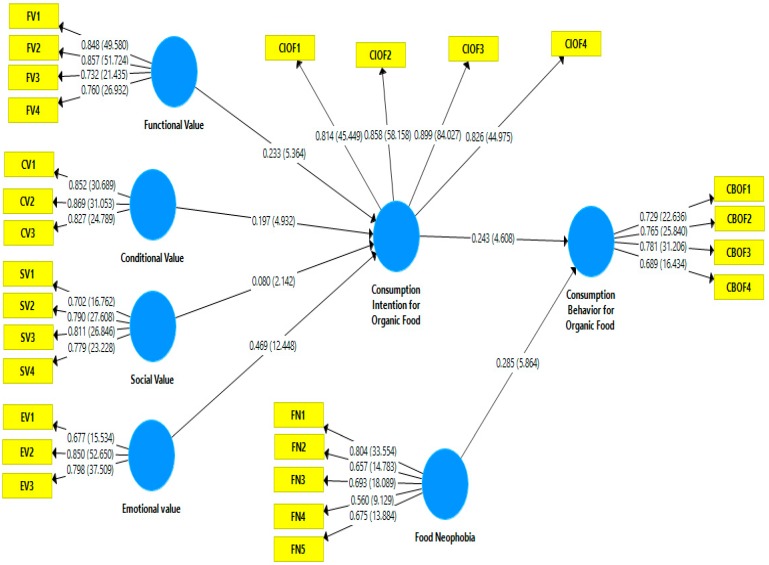
Structural model evaluation (Bootstrap).

Assessment of measurement model based on reliability (item reliability and internal consistency reliability) and validity tests (convergent validity and discriminant validity) [109]. Item reliability is measured by outer loading, inter consistency reliability is measured by composite reliability and convergent validity is measured by average variance extracted (AVE). As mentioned in Table 2, all items loading are well above the threshold value of 0.5 [110]. The composite reliability of each construct surpass the cut-off value of 0.7 and AVE exceed the recommended value of 0.5 [111]. The results show that all the AVE values are in between 0.566 (food neophobia) and 0.723 (Consumer intention and consumption behavior for organic food), all CR values are in between 0.820 (Emotional value) and 0.912 (Consumer intention for organic food) and all outer loadings are in between 0.5 and 0.9 (see Table 2 and Figure 2).

For discriminant validity, Heterotrait-Monotrait ratio of correlations (HTMT) test was used as it is robust than other methods. According to Kline [112], HTMT value should be less than 0.85, while Gold et al. [113] argue that this value should be less than 0.90 to confirm discriminant validity. All HTMT values are under the recommended threshold (see Table 3).

### 4.2. Structural Model

After completing the first stage of PLS-SEM i.e., assessment of measurement model, assessment of structural model was performed. Assessment of structural model based on path coefficients (β values), *t* values, effect size (f^2^), coefficient of determination (R^2^) and predictive relevance (Q^2^). Path coefficients significance was measured by the bootstrapping method (5000 re-sample). The results indicate that all six hypotheses are accepted (see Table 4, Figure 2) i.e., functional value (β = 0.233, *t* = 5.364 > 1.64, *p* < 0.05), social value (β = 0.080, *t* = 2.142 > 1.64, *p* < 0.05), emotional value (β = 0.469, *t* = 12.448 > 1.64, *p* < 0.05), conditional value (β = 0.197, *t* = 4.932 > 1.64, *p* < 0.05) are significant on purchase intentions of organic food. Table 4 shows that purchase intentions have a positive effect on purchase behavior with (β = 0.243, *t* = 4.608 > 1.64, *p* < 0.05), and food neophobia moderates the relationship of purchase intention and behavior (β = 0.080, *t* = 2.048 > 1.64, *p* < 0.05). R^2^ value for purchase intention is 0.493 and for consumption behavior is 0.222 which indicate that model has significant explanatory power for organic food consumption. However, supporting a model only based on R^2^ is not an effective approach [108]. Therefore, it is better to assess predictive relevance Q^2^ of the model. It is rule of thumb that if Q2 value is greater than 0 then latent exogenous constructs have high predictive relevance for latent endogenous constructs [108,114]. The value of Q^2^ is 0.328 for consumer purchase intention and 0.113 for consumer consumption behavior for organic food It suggests that model has high predictive relevance. f^2^ value is assessed as per Cohen [115] which indicate that f^2^ values 0.02, 0.15 and 0.35 are small, medium and large effect respectively. The values of f^2^ posit that size effect vary from medium to large effect.

### 4.3. The Moderating Role of Food Neophobia

**Figure 3:**
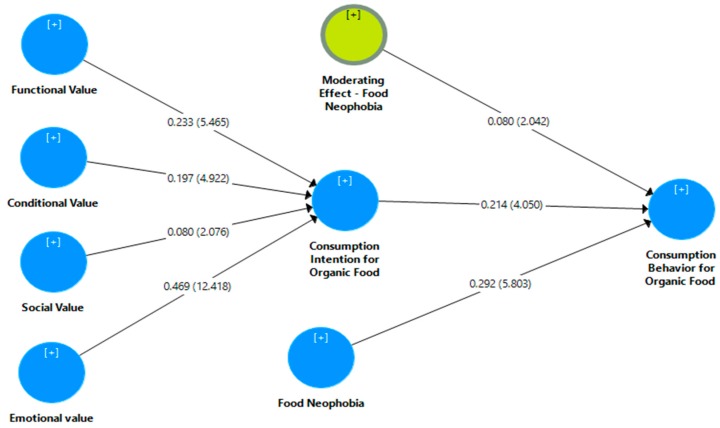
Moderating effect of food neophobia.

The moderating effect of food neophobia is examined by the interaction effect on the purchase intention and purchase behavior which can be seen in Figure 3. The results demonstrate that food neophobia significantly (β = 0.080, *t* = 2.048 > 1.64, *p* < 0.05) moderates the relationship between purchase intention and purchase behavior of organic food. This moderation has changed the coefficient of determination R^2^ of the model. The R^2^ value of purchase behavior has increased from 0.222 to 0.243. It means that after the inclusion of food neophobia, the model explains greater variation in the purchase behavior due to exogenous variables. Though, the difference in the variations is not very large yet still its plays a very significant role in analyzing the interaction and moderation effect.

## 5. Discussion

Unhealthy eating habits and the negative effects of conventional food (on the soil, environment and animals) have made consumers conscious about their food preferences and they are shifting rapidly towards organic food all over the globe. Despite this worldwide shift of food preferences, organic food adoption is found to be low in Pakistan. Therefore, the primary objective of this study was to identify the factors that affect consumers’ actual organic food buying behavior in Pakistan. In line with previous studies [23,30,56], we adopt the GPV model to find consumers’ intentions towards organic food and its subsequent effect on the actual buying behavior of organic food. Functional value, social value, emotional value, and conditional value were found to have significant and positive affect on consumer intentions. Moreover, the moderating role of food neophobia is also checked through intention behavior gap. Empirical results show that food neophobia moderates the relationship between consumer intentions and consumers buying behavior of organic food in Pakistan. The possible reasons for the results are discussed further.

Functional value is found to be a significant factor to positively affect consumer intentions to buy organic food with the (β = 0.233, *t* = 5.364 > 1.64, *p* < 0.05). The functional value is the very basic utility that a consumer desire from a product which in the case of organic food can be its quality, price, health benefits, and taste. The results of this study are in line with the study of Rahnama [6] who found that Iranian women consider functional value while consuming organic food. these results are also verified by the studies conducted in China and Taiwan [23,116,117]. The possible reasons for the result can be the price and health factors in Pakistan. The increasing ratio of NCDs is forcing consumers to change their food preferences and consumption patterns. Besides, the price of organic food is higher than conventional food in Pakistan which can be a reason for low adoption. As literature suggests that Pakistani consumers pay more attention towards price than quality to consume organic food [2].

Social values involve the perceived utility attained by the association of alternative capacity of consumers to buy products/services owing to social pressure, status and peer influence. H2 claims that social value has a significant positive effect on consumers’ intention to buy organic food which is supported by empirical result with (β = 0.080, *t* = 2.142 > 1.64, *p* < 0.05). These result are also in agreement with previous research on green consumerism conducted in Pakistan, India, Bangladesh and Portugal [2,3,118,119]. The possible reason for this result can be class consciousness, social status, and self-image of Pakistani consumers among friends, family, and another social group. Consumers in Pakistan especially young consumers care what people think about them, their consumption pattern, and their behavior.

Emotions are a necessary part of humans’ life and involve positive feelings like, excitement, joy, feeling of pleasure, enjoyment, doing good and negative feelings like anger, anxiety, fear, worry, and tension. Emotional value is the perceived utility obtained through the positive emotions of individuals derived by green consumption behavior. The results reflect that emotional value positively and significantly influence consumer intentions to buy organic food (β = 0.469, *t* = 12.448 > 1.64, *p* < 0.05). The results are in line with recent research of Woo and Kim [23] who found positive effect of emotional value on organic food consumption in china and verified the result of the studies conducted in Taiwan, Iran and USA [66,68,120] and have some contradictions with the results of India, Bangladesh and China [3,67,118]. The acceptance of H3 in Pakistan can be due to the fact that Pakistani consumers are emotional decision-maker rather than rational [75].

Conditional value refers to the perceived utility attain by the association of alternative capacity of a different situation. The results reveal that conditional value is positively associated with the consumer intention to consume organic food (β = 0.197, *t* = 4.932 > 1.64, *p* < 0.05). Previous research validate this result [2,4,6,23,66]. It means situations like discounts, promotions, incentives, easy availability of organic food can lead consumers towards organic food consumption. The results show that the availability of organic food can be one of the main factors behind the low adoption of organic food.

The study hypothesizes that consumers’ intentions positively and significantly affect consumers actual buying behavior of organic food which is accepted by the empirical results with (β = 0.243, *t* = 4.608 > 1.64, *p* < 0.05). These results has validation from some previous researches like Testa et al. found that consumer intention positively affect consumers actual buying behavior of organic food in Italy [61] which is the confirmation of the study conducted in China for green aquatic products consumption [101]. Though results indicate that only 22 percent of the total intended consumers lead toward actual buying behavior. This can be attributed to price and availability of organic food.

Empirical evidence reveals that food neophobia moderates the relationship of purchase intention and behavior of organic food. The results are in agreement with previous studies [35,84]. Food neophobia is reluctant, refusal or fear to use novel or unfamiliar foods. It means that individuals with high food neophobia involve less in organic food buying behavior in Pakistan and vice versa. This is also evident in previous studies which found that consumers avoid healthy eating (functional food) due to food neophobic personality trait [36,93]. The probable reasons for this result can be less knowledge of organic food. In Pakistan, organic food is at an introductory stage, which makes it unfamiliar to the general consumers. As recent study conducted by Rejmen [121] suggested that consumers are not familiar with sustainability in food choices and there is need of knowledge dissemination.

Based on the above empirical results, marketers should design flexible pricing strategies for organic food in a way that these prices are competitive with traditional food and provide the best value for money as consumers in developing countries search for the best value for money because the majority of the population belong to middle class families and have lower income [2,46]. Moreover, awareness of organic foods’ benefits as compare to traditional foods can be initiated to persuade consumers why organic food is more expensive than conventional food. Consumers like to perform those tasks which are encouraged by their social group, peer influence, and co-workers. Word of mouth regarding their behavior matters a lot for them. In this regard, electronic word of mouth campaigns can be launched by producers and food authorities to involve green consumers to motivate others in buying organic food. Positive emotions influence consumers to involve in organic food consumption as they feel good, satisfied, and relaxed, so marketers can also use emotional appeals in advertisements to promote organic food in Pakistan. An integrated message involving quality, price, social affiliation and emotional appeal can be conveyed by the food authorities to make the youth become involved in organic food consumption. In Pakistan, organic foods are only available at big and exclusive superstores [14]. Therefore, the easy availability of organic foods to every consumer could increase the consumption of organic foods. Moreover, promotion strategies, free samples of organic food of organic food products can also affect consumers’ intentions to buy organic food. Moreover, food authorities should ensure organic food is available at medium to large stores. In-store awareness programs can be initiated to make consumers involved in green consumption. Thus, food authorities can conduct seminars regarding the benefits of organic foods over conventional foods and should provide samples of these foods, so awareness regarding organic foods’ environmental and health benefits can be provided to general consumers as knowledge is very much important to reduce the fear of consumers. Moreover, marketers should use a push strategy at the beginning to maximize the market share of organic products so that consumers become aware of the benefits of organic foods.

## 6. Conclusions and Future Research Directions

The aim of the present study was to identify the factors that affect consumers’ actual buying behavior regarding organic foods. For this purpose, a theoretical foundation based on the GPV model was used to observe the actual buying behavior of Pakistani consumers. Moreover, the moderating role of food neophobia was also investigated. Data was collected from university students through valid questionnaires and PLS-SEM was employed to test the hypotheses. Results verify all six hypotheses proposed in this study and suggest that food neophobia is a factor that affects consumers’ intention and actual organic food buying behavior.

The SEM results show that functional value, social value, emotional value, and conditional value positively and significantly affect consumers’ intentions towards organic food consumption. The results are found to have similarities and disagreements with some previous studies due to differences in culture, context, and product category. In Pakistan, the price and availability of organic food are very crucial factors in organic food consumption. Organic foods are available at high prices and only at exclusive stores in big cities. Hence, setting competitive prices and enhancing availability to a maximum number of consumers can promote consumers’ intentions to consume organic food. With this strategy emotional and socially appealing advertisements can be used to motivate consumers as Pakistani consumers are inclined to engage in emotional decisions. Moreover, they consume products that are appreciated by others and increase their social or symbolic value. Furthermore, different incentives like promotions, discounts, and subsidies for organic foods can also increase their purchase intention. Moreover, our study shed light on the relationship between intention and behavior, which is less investigated in the organic food literature suggesting there is need to promote actual buying behavior. It is also observed that consumers’ actual buying behavior towards organic food can be increased by reducing the food neophobic personality elements of consumers. In this regard, the food authorities of all provinces (Punjab, Sindh, Baluchistan and Khyber Pakhtunkhwa) in Pakistan can play a major role. They can initiate an integrated communication program all around the country to render positive information regarding organic food such as awareness about health and environmental benefits of organic food. Likewise, the causes of NCDs like consumption of unhealthy food should also be emphasized in such campaigns. Moreover, consumers with high food neophobia will purchase organic food if organic food is proven safe and healthy through certification and health labelling by food authorities or government institutions. Therefore, food authorities can make sure that organic food is available at all levels of retail stores to make it accessible for general public.

### Limitations and Future Directions

Although, this study makes several empirical contributions and entails policy implications it still it has some limitations that can be addressed by future researchers. First, this study has considered general organic food consumption in Pakistan. Future studies in this area can examine the consumption of specific organic foods like organic milk, yogurt, cheese, vegetables, and fruits. Second, this research is limited to only Pakistani consumers; future research can undertake a cross-cultural sample for a deep insight into this phenomenon. Third, the study has only investigated the moderating role of one food personality trait i.e., food neophobia but moderating role of other food personality traits i.e., food involvement can be worth an exploration. Fourth, this study focuses only on millennials, future research can explore consumption patterns of a different age groups or demographic category.

## Figures and Tables

**Table 1:** Demographic profile.

	Characteristics	Frequency	Percentage%
Gender	Male	147	66.5
	Female	74	33.5
Age	18–21	21	9.5
	22–25	53	24.0
	26–29	88	39.8
	30–over	59	26.7
Education	Intermediate	40	18.0
	Undergraduate	102	46.2
	Graduate	58	26.3
	Professional	21	9.5
Household Income (PKR)	Less than 50,000 ($320)	54	24.5
	50,001 ($320)–75,000 ($480)	88	39.8
	75,001 ($480)–100,000 ($638)	39	17.6
	100,001 ($638)–125,000 ($797)	24	10.8
	125,001 ($797) and over	16	7.3

**Table 2:** Measurement Model Assessment.

Construct	Item	Loadings	AVE	CR
Functional Value	FV1	0.848	0.642	0.877
	FV2	0.857		
	FV3	0.732		
	FV4	0.760		
Social value	SV1	0.702	0.595	0.854
	SV2	0.790		
	SV3	0.811		
	SV4	0.779		
Emotional Value	EMV1	0.677	0.606	0.820
	EMV2	0.850		
	EMV3	0.798		
Conditional value	CDV1	0.852	0.722	0.886
	CDV2	0.869		
	CDV3	0.827		
Consumer Intention for Organic Food	CIOF1	0.814	0.723	0.912
	CIOF2	0.858		
	CIOF3	0.899		
	CIOF4	0.826		
Food Neophobia	FN1	0.804	0.566	0.811
	FN	0.657		
	FN	0.693		
	FN	0.560		
	FN	0.675		
Consumption Behavior for Organic Food	CBOF1	0.729	0.723	0.830
	CBOF2	0.761		
	CBOF3	0.781		
	CBOF4	0.689		

**Table 3:** Discriminant validity (HTMT).

	CV	CBOF	CIOF	EMV	FN	FV	SV
**CV**							
**CBOF**	0.197						
**CIOF**	0.314	0.512					
**EMV**	0.133	0.597	0.771				
**FN**	0.204	0.573	0.738	0.792			
**FV**	0.188	0.473	0.620	0.661	0.739		
**SV**	0.067	0.352	0.394	0.457	0.466	0.479	

**Table 4:** Structural Model Results (Hypotheses testing).

Hypothesis	Relationship	Path Coefficient	Std. Error	*t* Value	*p*-Value	Supported	R^2^	Q^2^	f^2^
H1	FV -> CIOF	0.233	0.043	5.364	0.000	Yes	0.493	0.328	0.073
H2	SV -> CIOF	0.080	0.037	2.142	0.016	Yes			0.010
H3	EMV-> CIOF	0.469	0.038	12.448	0.000	Yes			0.315
H4	CDV -> CIOF	0.197	0.040	4.932	0.000	Yes			0.074
H5	CIOF -> CBOF	0.243	0.053	4.608	0.000	Yes	0.222	0.113	0.049
H6	Moderating effect FN -> CBOF	0.080	0.036	2.042	0.000	Yes	0.243		0.068

## Appendix A

### Appendix A.1. Functional Value

FV1: Organic foods are good products for the price.
FV2: Organic foods are economical for the attributes they offer.
FV3: Organic foods have an expectable standard quality.
FV4: Organic foods are made from non-hazardous Substances.

### Appendix A.2. Social Value

SV1: Buying organic food would help me to feel acceptable.
SV2: Buying organic food would improve the way that I am perceived.
SV3: Buying organic food would make a good impression on other people.
SV4: Buying organic food would give its owner social approval.

### Appendix A.3. Emotional Value

EV1: Buying organic food instead of conventional food would feel like making a good personal contribution to something better.
EV2: Buying organic food instead of conventional food would feel like the morally right thing.
EV3: Buying organic food instead of conventional food would make me feel like a better person.

### Appendix A.4. Conditional Value

CV1: I would buy organic food instead of conventional food under worsening environmental conditions.
CV2: I would buy organic food instead of conventional food when there is a subsidy for green products.
CV3: I would buy organic food instead of conventional food when there are discount rates for green products or promotional activity.
CV4: I would buy organic food instead of conventional food when green products are available.

### Appendix A.5. Purchase Intention for Organic Food

CIOF1: I would like to buy organic food products to reduce environmental damage.
CIOF2: I would like to buy organic food products to guarantee my health.
CIOF3: As much as possible, I plan to buy organic food products.
CIOF4: I am willing to pay more for organic food products.

### Appendix A.6. Consumer Behavior for Organic Food

CBOF1: How often do you buy organic food products?
CBOF2: I always try to buy organic food with green labeling marks.
CBOF3: I buy organic food products even if they have a higher price.
CBOF4: I recommend organic food products that I consume to my relatives and friends.

### Appendix A.7. Food Neophobia

FN1: I do not trust new foods.
FN2: I am constantly sampling new and different foods.
FN3: I am afraid to eat things I have never had before.
FN4: I will eat almost anything.
FN5: If I do not know what is in a food, I won’t try it.
